# Using Best–Worst Scaling Survey to Investigate the Relative Importance of Attributes Associated with Public Hospital Outpatient Appointments

**DOI:** 10.1007/s40271-025-00732-y

**Published:** 2025-02-26

**Authors:** Tilley Pain, Amy Brown, Gail Kingston, Stephen Perks, Corey Patterson, Nerida Firth, Jessica Lester, Luke Sherwood, Sonja Brennan, Deborah Street

**Affiliations:** 1 Allied Health Governance Office, Townsville Hospital and Health Service, Townsville, QLD Australia; 2 College of Public Health, Medical and Veterinary Sciences, James Cook University, Townsville, QLD Australia; 3 Townsville Cancer Centre, Townsville Hospital and Health Service, Townsville, QLD Australia; 4 College of Medicine and Dentistry, James Cook University, Townsville, QLD Australia; 5 Townsville Pharmacy Department, Townsville Hospital and Health Service, Townsville, QLD Australia; 6 Allied Health Services Division, Townsville Hospital and Health Service, Townsville, QLD Australia; 7 Alcohol, Tobacco and Other Drug Service, Townsville Hospital and Health Service, Townsville, QLD Australia; 8 Clinical Governance Directorate, Townsville Hospital and Health Service, Townsville, QLD Australia; 9 North Queensland Maternal Fetal Medicine Unit, Townsville Hospital and Health Service, Townsville, QLD Australia; 10 Centre for Health Economics Research and Evaluation, University of Technology, Sydney, Australia

## Abstract

**Introduction:**

Obtaining patient input before healthcare redesign improves patient experience. The Townsville Hospital and Health Service, a regional Australian public health service, seeks to reduce the long wait list for medical specialist appointments by introducing allied health substitution models of care for low-acuity patients. This paper describes a best worst scaling survey conducted to refine attributes associated with outpatient appointments which will be used in a future discrete choice experiment (DCE).

**Methods:**

A literature review was conducted to identify attributes associated with medical specialist outpatient appointments and allied health substitution models. An object (or case 1) best worst scaling (BWS) survey was designed using blocks of a balanced incomplete block design and analysed using multinomial logit and mixed logit models. Patients waiting at local specialist outpatient clinics were invited to complete the survey via an iPad. The interviewer collected field notes, which were analysed using content analysis.

**Results:**

A total of 12 attributes were identified in the literature review and one from local discussion. The 167 completed responses demonstrated the ranking of attributes were diagnostic accuracy, symptom relief, continuity of care, satisfaction with care, healthcare professional, manner and communication, time on waitlist and onward referral. The least important attributes were reassurance offered, appointment wait time, cost and appointment duration.

**Conclusions:**

This BWS survey allows us to reduce the attributes for inclusion in the DCE from 13 to 8. Diagnostic accuracy and symptom relief were of most importance, and appointment wait time and duration were of least importance. This suggests that patients would be willing to be attend different models of care such as allied health primary contact model if clinical outcomes were equivalent to the current medical-led models.

**Supplementary Information:**

The online version contains supplementary material available at 10.1007/s40271-025-00732-y.

## Key Points for Decision Makers

**Table:** 

BWS surveys are robustly designed surveys that can be used to elicit the relative importance patients place on attributes associated with aspects of healthcare prior to developing or changing clinical service.
BWS surveys can be used to refine/reduce the number of attributes thereby reducing the attribute burden when designing a discrete choice experiment.
Patients’ responses indicate they place the highest importance on diagnostic accuracy, symptom relief and continuity of care when attending medical specialist appointments at a public health service.

## Introduction

Long waiting times for public outpatient medical specialist clinics are a challenge internationally. [1] In Australia, patients are referred by general practitioners in primary care to be seen by public or private medical specialists. Referral requests sent to public hospitals are triaged into three levels, depending on acuity: category 1 has a recommended maximum wait time of 30 days, category 2 has a wait time of 90 days and category 3 has a wait time of 12 months. Often, patient wait times exceed the recommendations due to various factors relating to poor resource alignment, operational inefficiencies and limited process improvement strategies [2]. Additionally, delays in access to medical specialists can distress some patients and result in worsening clinical outcomes [2, 3]

Numerous strategies have been identified to reduce the long wait times for outpatient appointments. One strategy is allied health substitution models, in which professions such as occupational therapy, physiotherapy, dietetics, speech pathology and podiatry can streamline low-acuity patients via screening clinics [4]. In acute or secondary healthcare, models in which allied health professionals substitute for medical specialists are also referred to as allied health primary contact models [5]. If patients receive an initial assessment and treatment by an allied health professional, they can be removed from the waitlist once they are successfully treated, reducing the waitlist size. Implementing allied health primary contact models has reduced wait times, expedited referral of appropriate patients to surgery [6] and demonstrated more effective utilisation of medical specialists [2]. Allied health primary contact models have been shown to provide safe, effective and timely care [7], suggesting that implementing additional models could benefit patients and the health service.

Health service delivery in the aftermath of the coronavirus disease 2019 (COVID-19) pandemic has changed substantially [7]. The pandemic caused the cancellation of many elective surgeries, exacerbating the long wait lists for specialist outpatient referrals in Australian public hospitals. Several Australian health services are exploring ways to reduce the number of patients waiting for a medical specialist appointment [8]. Townsville Hospital and Health Service (THHS) utilise allied health substitution models of care, but other strategies could be implemented to reduce waitlists further. To date, only one study has explored patient preference in gastroenterology substitution, showing that patients have a strong but varied preference for gastroenterology services depending on their prior exposure to allied health substitution and expanded scope models of care [9]. Another study demonstrated the dimensions of healthcare delivered by allied health professionals that matter to patients were balancing expectations, timely access and convenience and continuity of care [10]. However, little is known about patient preferences in other geographical areas of Australia or other substitution models, indicating a gap in evidence needing to be addressed before the implementation of new models of care. Before introducing allied health substitution models, decision-makers need to investigate the preferences of those likely to be affected by the proposed change—patients and medical specialists. A discrete choice experiment (DCE) is a rigorous and appropriate method for obtaining preferences.

A DCE can be used to acquire information about patient preferences regarding attributes associated with health service delivery. The patient-acquired information can be used to inform decision-makers responsible for healthcare design. A DCE was used in a previous study to determine patient preferences for specialist gastroenterology services, including questions to elicit the preference of the primary treating healthcare professional [10]. Selecting attributes to include in the DCE can be done via qualitative data collection [11], including a literature review. Once identified, attributes for inclusion into a DCE can be refined by conducting a best–worst scaling (BWS) survey [12].

We propose to use an object (or case 1) BWS survey to ensure attributes that patients considered of high importance are included in the DCE [13]. The BWS survey uses a master list of items from which a specified cohort can identify the most and least important items via completion of the BWS survey. The BWS protocol includes showing each respondent a subset of items from the master list and asking them to indicate the ones that they think are the most and the least important. By allowing the selection of the most and least important attribute, participants are trading off the other attributes within the subset, allowing for calculation of the relative importance of the attributes [14]. Each respondent chooses the most and least important item repeatedly, with the subsets of items in each set varying according to a statistical design. The resultant ranking of attributes allows for the selection of the most important attributes, as well as which are also relevant to the topic, to reduce the number of attributes carried forward to the DCE, thereby reducing the cognitive burden on respondents.

## Methods

### Context

This study was conducted at the Townsville University Hospital (TUH), a regional tertiary hospital serving a large, geographically dispersed population of northern Queensland. TUH currently has three substitution models for physiotherapy: neurosurgical and orthopaedic, urogynaecology and vestibular screening clinics. A dietetics substitution model in gastroenterology was discontinued and a speech pathology substitution model is in planning. Numerous substitution models have been described [4], and the THHS Executive Director of Allied Health (EDAH) is motivated to explore patient perspectives through this study and subsequent works with the view of expanding substitution models within the health service. The research team included allied health clinicians involved in substitution models (C.P., J.L., L.S.) and others with subject matter expertise and interest in BWS and DCE (D.S., A.B., N.F.).

### Allocation Principles

Attributes were identified via a literature review using the Population, Concept and Context (PCC) structure proposed for scoping reviews. For this study, the population referred to people waiting for a medical specialist appointment at a public hospital, the concept was allied health substitution models and the context was public hospital outpatient appointments to any medical specialty. The research team followed the steps described by Arksey and O’Malley (2005) [15] to ensure robustness of the review. First, the research team developed the research question, “What is known about patient preferences for allied health substitution models in public hospital medical specialist clinics?” Second, one member of the research team with a full-time research role (TP) consulted with a librarian and searched electronic databases MEDLINE, CINAHL, EMBASE, Cochrane Library and Health Policy Reference Centre using search terms agreed upon by the research team. Synonyms of all search terms (e.g. physiotherapist, physical therapist) were identified by conducting repeated searches of the listed databases. The databases were then searched using a combination of key words and MeSH terms for (specialist referral OR specialist consultation OR specialist pathway), and (substitution OR delegation OR expanded scope) and (allied health OR health professional). Third, studies identified during the search were screened using the title and abstract to select full text articles. One researcher (TP) reviewed all full-text articles, and the role of the second reviewer was divided equally among the other team members (including clinician researchers). Papers were excluded unless they referred to allied health substitution models for medical specialists in public hospital outpatient settings. A data synthesis strategy was developed to overcome the heterogeneous nature of the included full text papers. This included a narrative summary for submission to the THHS Executive. Critical appraisal skills were used rather than appraisal tools to describe the level of evidence on the basis of the NHMRC hierarchy, as critical appraisal is not a requirement of scoping reviews [15]. The findings were aggregated into a table (Supplement 1) to identify criteria relevant to allied health substitution models. The criteria were then presented to the research team, including the clinicians working in substitution roles, for discussion and relevance to the BWS survey question until the team unanimously agreed on the attributes to be included (Table 1).

**Table 1:** List of attributes, the reference from which it was derived collated from the literature review or subsequent discussion with the investigators and the descriptor provided to participants

Characteristic	Reference	Descriptor
Type of health professional		On attending the hospital, you may see a variety of health professionals including medical specialist, medical registrar, allied health professional, nurse or nurse practitioner
Time on wait list		The National Standard’s waiting time for patients triaged in categories 1, 2 and 3 are 1, 3 and 12 months, respectively. However, sometimes you may be seen in less than the nominated wait time or more than nominated wait time
Appointment duration	[9]	Depending on the complexity of your case, you may be with the health professional for between 10 and 60 min
Onward referral	[25, 30–35]	Following the screening by the health professional, you may be referred to another service or health professional for treatment or further investigations
Continuity of care	[9, 25, 33]	If you attend the clinic often you may see the same or different health professionals
Diagnostic accuracy	[6, 25–29, 36]	While every effort for accuracy is taken, there may be variations in the accuracy of the diagnosis
Cost	[9, 36–43]	Public health hospitals provide free services in Australia. Private appointment requires out-of-pocket costs and may be seen earlier
Symptom relief	[33, 44]	Patients may experience relief of their symptoms from an intervention by the health professional
Manner and communication	[9]	The manner and communication skill of the healthcare professional treating you
Reassurance offered	[9]	The healthcare professional may provide reassurance that your symptoms are not because of a more serious or life-threatening condition
Satisfaction	[27, 42, 45–50]	Whether you are satisfied with the care you are provided
Location of appointment	Expert addition for local context	Health services can be provided at different locations such as the hospital, Kirwan, North Ward or Cambridge Street Community Health Campuses
Appointment wait time		Upon arrival at the hospital, you may have to wait to see the health professional. Wait time may be as short as 10 min to 2 h or more

### Best–Worst Scaling (BWS) Survey Design

This study used a BWS survey in which respondents were presented with 13 sets of 4 items. Participants selected their choice for the most important and least important characteristic from those presented in each choice set (see Supplement 2 for Patient Survey). Each choice set contained four items, and across the 13 choice sets seen by each respondent, each item appeared in exactly four choice sets and each pair of items appeared in exactly one choice set. Thus, the 13 choice sets shown to each respondent were the blocks of a balanced incomplete block design (BIBD), the most common approach to designing BWS surveys [16]. We used three different BIBDs for data collection, as the work by Furlan and Turner suggest that more versions lead to better results [17].

### Population

Patients in the waiting room of TUH specialist outpatient clinics during March and April 2024 were the target patient population for the BWS survey. This population was chosen to obtain information of specific relevance to this health service in which the substitution models are proposed to be introduced. Using a convenience sampling approach, patients waiting for surgery, cardiac, and ear, nose and throat (ENT) outpatient appointments were approached by one of the researchers (T.P.) and asked if they would participate. On affirmation, patients were given an iPad to complete the survey electronically, with consent implied through the completion. The researcher helped patients who asked for assistance to use the iPad to complete the survey. The researcher exercised caution when assisting people with the technology to avoid influencing their responses. To ensure consistent messaging, a description of the attributes was printed and given to participants to answer queries about the attributes (Table 1). With only four attributes per question, the research team decided a priori for one ‘best’ and one ‘worst’ selection only per question, otherwise the response would be deemed incorrect and removed from the analysis. The survey was emailed to medical specialists at the local health service as the other stakeholder group of interest in introducing substitution models.

Currently, there is no formal guidance on the optimal sample size for BWS [18], with sample size varying widely between 100 and 300 in a review of 56 DCE studies [19], with a suggestion that the sample size for BWS cannot be estimated a priori [20, 21]. Ultimately, the sample size was a pragmatic decision based on patients’ willingness to respond and the cost of administering a face-to-face survey. Therefore, when the survey tool (REDcap) indicated > 250 completed responses, data collection was stopped [19]. As the survey was approved by ethics for low-risk data collection, it meant no identifying information could be collected on the number approached or why people chose not to participate. Therefore, no response rate can be calculated.

### Pre-testing of Survey

The survey was tested firstly amongst the research team, and then in a volunteer group of eight people. The volunteer group were asked to conduct the survey and provide feedback. A statement describing the seeming similarity of choice sets with assurance that the attributes do change was added at this stage. Another minor change was to provide the Mutsekwa et al. (2023) description for ‘manner and communication’ in the handout to patients [9].

### Statistical Analysis

Statistical analysis was performed in the R software package [22, 23]. Count analysis and the mixed logit (MIXL) were used for analysis in this study on the basis of the observations in Cheung et al. (2019) [16]. These authors compared various analysis methods proposed in the literature, including count analysis, MNL, mixed logit, latent class analysis and hierarchical Bayes estimation. They concluded that the five statistical methods yielded similar rankings, particularly for the top few and bottom few items. They also noted that by using latent class or mixed logit models, it was possible to comment on whether there was heterogeneity among patients for the items presented. Hence, the MIXL was the model estimated in this study. The attributes in the BWS survey do not have levels, thus the analysis assumes that each respondent makes choices according to a latent scale, typically seen as ‘utility’. However, it might also be considered as ‘degree of concern’ depending on the type of attribute used. An introduction to BWS surveys can be found elsewhere [24].

Participant characteristics and demographics were analysed using descriptive statistics for both complete and incomplete survey responses. Comparisons between the groups was conducted using chi-squared and Mann–Whitney *U* tests.

### Field Notes

The researcher conducting the face-to-face surveys recorded field notes during the data collection period. Field notes included overviews of discussions held between the researcher and patients, as well as observations made by the researcher during data collection in the outpatient clinics. No field notes were linked to individual participant responses. Content analysis of the field notes identified the most frequent comments.

### Ethical Considerations

This project was submitted to the THHS Human Research and Ethics Committee and considered as meeting the classification of non-research by the Audit, Quality and Innovation Review Panel (THHSAQUIRE 1667).

## Results

### Literature Review

A total of 45 studies were identified as relevant to allied health substitution for medical specialists in public hospital outpatient clinics. Most studies were of low-level quality using the NHMRC Evidence Hierarchy. All papers were from countries with universal healthcare: Australia (*n* = 17), United Kingdom (*n* = 13), Canada (*n* = 7), Sweden (*n* = 1) and New Zealand (*n* = 1). The earliest publication was from 1994 and the latest from 2021, with most (*n* = 32) published since 2012. The studies described substitution models for ten medical specialties and nine allied health professions.

A total of 12 attributes were identified from the literature review, and an additional attribute arose from discussion amongst the investigators on the basis of relevance to the local population. A previous DCE on substitution models in gastroenterology care identified seven attributes from the literature and patient interviews [9]. Additional attributes were predominantly used as outcome measures in studies describing allied health substitution models. For example, diagnostic accuracy was included, as several studies compared the diagnostic accuracy of allied health professionals to medical specialists [6, 25–29]. The attributes included in the BWS survey are listed in Table 1.

### Demographics and Characteristics

A total of 279 patient surveys were commenced, with 112 excluded due to being incomplete (*n* = 106) or completed incorrectly (*n* = 6, see Table 2). The predominant reason for being incomplete was that patients were called in for their appointment prior to them completing the survey. Despite help being offered by the survey administrator, some respondents were not receptive and failed to see that the attributes available for selection varied from set to set. Due to the level of ethics approval obtained, no attempt was made to obtain a reason for patient’s refusal to participate. A total of 38 medical specialist surveys were commenced, with 16 excluded as incomplete or completed incorrectly. Due to insufficient medical specialist responses, only patient demographics and preferences will be presented.

**Table 2:** Breakdown of number of survey commencement, completions and dropouts.

	*n*	% of commencements
Commenced the survey	279	100
Incomplete after initial demographics questions	34	12.2
Incomplete during BWS choice sets	72	25.8
Marked as complete but not all choice sets answered	6	2.2
All choice sets completed	167	59.8

Age ranged from < 20 years to > 80 years, with most participants falling within the 61–70-year-old age group. There was a similar proportion of male and female respondents, and most (43%) patients had been waiting for their appointment for less than 1 month (Table 3). No significant differences were found between included and excluded participants for gender. For appointment wait times, while the overall distribution did not differ significantly between groups (*p* = 0.051), individual category comparisons showed a higher proportion of included participants reported wait times of less than 1 month (43.1% versus 27.7%, *p* = 0.011). All other wait time categories showed no significant differences.

**Table 3:** Demographics of patients who did and did not complete the BWS survey and *p*-value comparing the two sets.

	Included (*n* = 167)	Excluded (112)	*p*-Value
Age (*p* = 0.6984)	Freqs (%)		
< 20 years old	10 (6.0%)	10 (8.9%)	0.53
20–30 years old	19 (11.4%)	13 (11.6%)	0.11
31–40 years old	19 (11.4%)	12 (12.7%)	0.57
41–50 years old	23 (13.8%)	13 (11.6%)	0.29
51–60 years old	25 (15.0%)	15 (13.4%)	0.21
61–70 years old	40 (24.0%)	20 (17.9%)	0.01
71–80 years old	27 (16.2%)	23 (20.5%)	1.00
> 80 years old	4 (2.4%)	6 (5.4%)	0.10
Gender (*p* = 0.5957)	Freqs (%)		
Female	81 (48.5%)	53 (47.7%)	1.00
Male	86 (51.5%)	58 (51.4%)	1.00
Non-binary	0 (0%)	1 (0.9%)	0.40
Appointment wait length (*p* = 0.0513	Freqs (%)		
< 1 month	72 (43.1%)	31 (27.7%)	0.01
1–2 months	36 (21.6%)	23 (20.5%)	0.88
3–5 months	27 (16.2%)	21 (18.8%)	0.63
6–11 months	12 (7.2%)	8 (7.1%)	1.00
1 year	4 (2.4%)	8 (7.1%)	0.07
> 1 year	13 (7.8%)	16 (14.3%)	0.11
Not sure	3 (1.8%)	5 (4.5%)	0.27

### BWS Coefficients

**Fig. 1:**
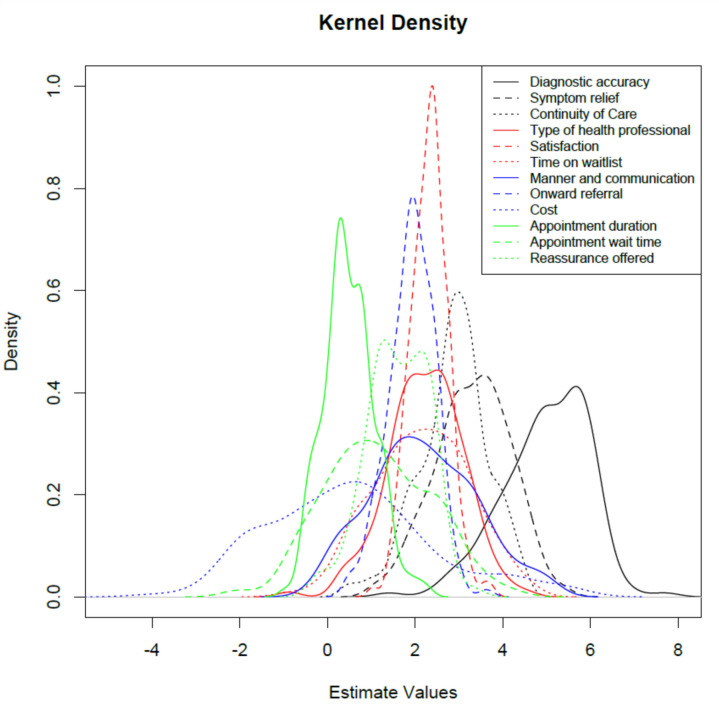
Kernel density plots of the estimated values from the MIXL analysis showing the distribution of the ranking of all attributes.

Analysis of the 13 attributes used to describe appointments in an outpatient setting were ranked in order of importance to patients (Table 4). The order of the attributes from most to least important was diagnostic accuracy, symptom relief, continuity of care, satisfaction with care, time on wait list, healthcare professional, communication, onward referral, reassurance offered, appointment wait time, appointment duration, cost and location. Although cost was of low importance, its distinguishing feature was its variability. Kernel density plots of the estimated values from the MIXL analysis show the density for each parameter (Fig. 1). Location of appointment was used as the reference as it was rated as least important by patients.

**Table 4:** Results from the MIXL analysis of the BWS survey listing the attributes from most important to least important

Attribute	Mean estimate	Standard error	SD estimate	SD standard error
Diagnostic accuracy	5.03514	0.19952	1.30761	0.16900
Symptom relief	3.24636	0.14782	1.16985	0.14015
Continuity of care	2.84108	0.14044	1.02907	0.12108
Satisfaction	2.34960	0.12674	0.66592	0.11858
Time on wait list	2.22862	0.15192	1.30529	0.12917
Type of health professional	2.19627	0.13888	1.09354	0.12285
Manner and communication	2.10847	0.15043	1.47911	0.13269
Onward referral	1.89440	0.12459	0.79133	0.12933
Reassurance offered	1.66504	0.12977	0.96207	0.11932
Appointment wait time	1.29356	0.14958	1.48445	0.13770
Appointment duration	0.51789	0.12341	0.86077	0.15521
Cost	0.43190	0.18213	2.04431	0.16284

The ranking of attributes was relative to ‘location of appointment’. Model performance statistics: log-likelihood −3965; number of observations 2171; AIC 7977.94; BIC 8114.33

### Field Notes

The field notes were classified into two broad categories: (1) patient’s comments or questions and (2) researcher’s observations. Listed below are the comments most frequently stated by patients.

Cost: Patients’ comments varied considerably when referring to cost. Some stated it was important because if they had to pay, they could not afford the treatment they need. Others stated it was not important because provision of healthcare is free in public health services in Australia.

Type of health professional: Patients considered seeing a health professional was important in two ways. First, some thought it important to see the most appropriate health professional regardless of discipline. A few considered it important to see a specific type of health professional, with most of those wanting to see the medical specialist they had been referred to.

Time on waitlist: Some confusion existed in answering this question. Some participants thought it referred to time they spent in the waiting room rather than the time since referral from their general practitioner (GP). However, when clarified it was from the time of GP referral, many of the participants stated they had regular appointments at the clinic.

Location: No participant stated they were concerned about where in Townsville the appointment was held. Participants who travelled from rural or remote locations stated the distance within Townsville to get to an appointment was less than the distance they had already travelled. Therefore, location was not at all important to rural and remote patients.

Observations by the survey administrator: Many participants found the BWS survey confusing, with patients reacting positively or negatively. Some stated it did not make sense and either did not finish it or ticked everything, whereas others asked how to do it, what it meant and what would be done with the results. Some participants surveyed in the surgical outpatient clinic had been recently discharged from the hospital with recent surgical scars, and many stated they were in a lot of pain but were required to attend for specialist review. The few who felt unhappy about their service described poor clinical outcomes, mix-ups with appointments and times, and perceived unprofessional behaviour.

## Discussion

This study identified and refined attributes for inclusion in a future DCE. A literature review and expert discussion were used to identify the attributes for inclusion. A total of 12 attributes were identified from the literature, and one attribute from expert discussion was relevant to the local context. These 13 attributes were included in the BWS survey to reduce the attribute selection and thereby reduce the cognitive burden of the proposed DCE. The eight attributes rated by patients as most important by the BWS survey will be included in the DCE.

BWS surveys are now commonly used to prioritise objects in health [51]; however, best practices for their use are still emerging [13]. Only one study has previously used BWS surveys to develop attributes before conducting a DCE. In it, the authors recommend selecting attributes using four criteria: they are rated as highly important to participants, address the research question, form coherent and realistic descriptions of the subject and the number should be small enough so that the cognitive burden on participants is reasonable. However, they did not use the most important rated attributes in the DCE [12]. In the current study, the top eight rated attributes demonstrating relative priorities of competing preferences of patients at TUH also meet the other three criteria suggested by Webb et al. (2021). The attributes omitted are those rated as a lower priority to patients at the health service at which the allied health substitution is proposed, and we argue, can be left off to reduce the number of attributes progressing to the DCE [14]. Attributes rated as important in the context or geographical location increase the relevance of the preferences identified by the DCE to the site at which the decisions will be made. For example, there were differences in attribute ratings between this study and the preferences identified in gastroenterology substitution, suggesting these differences may be due to context or geographical differences [9]. Furthermore, in this study, the criteria for selecting attributes proposed by Webb et al. (2021) were all met with inclusion of the top-ranked attributes from the BWS.

Diagnostic accuracy was ranked as the most important to patients in this BWS survey. Diagnostic accuracy was a common outcome measure in the literature review articles, usually assessed by comparing the diagnosis of the allied health professional with that of the medical specialist [6, 25, 26, 28, 29, 40, 42, 52]. The next two highest ranked attributes were symptom relief and continuity of care. Field notes suggested that surgical patients presented to the outpatient clinic soon after discharge, with many indicating they were in pain. Therefore, symptom relief may have ranked highly because of the high number of recent surgical patients surveyed. It is striking that the two clinical outcome attributes were chosen within the top three most important attributes, and it would be interesting to determine whether patients may be willing to accept worse levels in factors rated as less important, such as wait times as a trade-off for the best clinical outcome.

A previous DCE examining patient preferences for healthcare professional in gastroenterology clinics demonstrated an overwhelming importance of manner and communication [10]. In our BWS survey, manner and communication had a lower ranking of importance, as did satisfaction with care, healthcare professional, time on waitlist and onward referral. The type of health professional came through in the field notes, particularly in meeting patient expectations to see a medical specialist. Previous allied health substitution models of care found that patient attendance for allied health clinics was influenced by the wait time for the specialists—that is, if the wait list for the specialist was not long, they would opt not to access the allied health service [4]. While this could not be further examined in our present BWS survey, it will be examined in our DCE.

The remaining attributes included in this BWS survey showed no clear importance to patients. The attributes were location (used as the base attribute), reassurance offered, appointment wait time, cost and appointment duration. Appointment duration was preferred by patients waiting for a gastroenterology clinic in another study [10], but not mirrored by our results. The difference between the current and former studies on appointment duration was not explored. This will not be included in the DCE due to the lack of importance demonstrated by local patients. Patients were not asked if they had previous experience with allied health substitution models, which influenced responses in a prior study [10]. Field notes suggest patients were willing to see the most appropriate health professional for their needs. It would be interesting to compare responses of patients who have and have not experienced substitution models in a future study to elicit whether exposure was a greater motivating factor than wait time. There was a difference in the time on the wait list between included and excluded patients. The shorter wait time reported by included respondents suggests a high proportion of patients presenting for review and may have influenced the ranking of this attribute. Time on wait list is central to the question pertaining to allied health substitution models. It will be included in the DCE with the inclusion of additional clarifying questions to differentiate new and review appointments. This difference in baseline characteristic may have influenced the lower rating of this attribute by respondents.

The cost attribute elicited one of two comments from patients, as described in the field notes. On the one hand, cost was not important because the public health service was fee-free for outpatients. On the other, it was important because if there were out-of-pocket costs to the patient, then they could not afford to attend. In Mutsekwa [10], patients were averse to out-of-pocket costs. The overall aim of obtaining patient’s preference is to determine whether they are willing to see an alternative health professional if it will reduce their wait time. The time on waitlist attribute in the planned DCE will allow us to calculate willingness to wait. Therefore, we will not include the cost in the DCE to calculate willingness to pay, given the context of interest is a local practice change in a fee-free public health system.

### Specific Learnings from the BWS Survey to Incorporate Within the Planned DCE

The demographic wait-time question ‘Approximately how long have you been waiting for today's appointment?’ caused confusion and misinterpretation. Therefore, the question in the DCE will be reworded to ‘Approximately how long have you been waiting for a specialist appointment since the initial referral (from your GP or other doctor)?’ The DCE will specifically invite all outpatients who are currently on the waitlist for the hospital with the expectation that patients waiting longer than the recommended wait times will be captured.

Certain demographics may influence patient preferences, for example, employment/other duties that are disrupted to attend hospital appointments. While we kept the demographics/characteristics purposefully broad for the BWS survey, we will ask more refined questions for the DCE to allow for a more in-depth analysis of patient preferences. We will also have some basic demographics/characteristics of the invited population (including age, gender, wait-list category, wait-list sub-speciality) to compare with responders.

### Strengths and Limitations

The strength of this study is the contribution to the evidence and rationale for using BWS surveys to refine attributes for a DCE. This study adds further guidance to the methodological use of BWS surveys in developing attributes, suggesting a potential way to include nuances about context or geographical differences to refine attributes. Another strength is the inclusion of clinical staff involved in substitution models in the research team [24, 51].

The lack of stakeholder qualitative input into attribute selection is a limitation of this study that we attempted to address by including clinicians in the research team. Not identifying whether respondents were attending a new or review appointment is a limitation of this study and may have impacted perceptions of key attributes such as wait time. The total number of participants invited to participate in the survey was not recorded due to the level of ethics approval obtained. This is a limitation of the survey, as the total response rate cannot be calculated. Selection bias cannot be ruled out, as the researcher conducting the face-to-face surveys was limited to people waiting for an appointment in three specialist clinics’ waiting rooms. Sampling bias may have occurred, as a comparison between the characteristics of completed and non-completed participants shows a difference in wait time, with excluded participants having a longer wait time. This difference may have influenced the response to wait time and will be addressed in the DCE. Lack of input from other key groups, such as medical specialists, may mean the selected attributes do not fully reflect the priorities of all stakeholders involved in outpatient care given the difference demonstrated in another study [14]. However, expert opinion (and specifically medical specialists) will be sought prior to finalisation of DCE attributes. A final limitation is that a systematic review method was not used for the literature review.

## Conclusions

This BWS survey has reduced the attributes from the master list of 13 to 8 attributes of importance to carry forward into a DCE. For our population, these are: diagnostic accuracy, symptom relief, continuity of care, satisfaction with care healthcare professional, manner and communication, time on waitlist and onward referral.

The clinical outcomes of diagnostic accuracy and symptom relief were of the most importance, and logistics, such as appointment wait time and duration, were of the least importance. This suggests that patients would be willing to attend different models of care, such as an allied health primary contact model, if clinical outcomes were equivalent to the current medical-led models. Further evidence is required around broad stakeholder impact. Patient preferences will be further examined in the planned DCE study.

## Supplementary Information

Below is the link to the electronic supplementary material.

Supplementary file1 (XLSX 33 kb)

Supplementary file2 (DOCX 261 kb)

Supplementary file3 (DOCX 24 kb)

## Declarations

### Data availability

The data that support the findings of this study are available from the authors upon reasonable request.

## References

[1] Howell E. National wait times database needed. CMAJ Can Med Assoc J. 2008;178(2):139. 18195279 10.1503/cmaj.071777 PMC2175011

[2] Naiker U, et al. Time to wait: a systematic review of strategies that affect out-patient waiting times. Aust Health Rev. 2018;42(3):286–93. 28355525 10.1071/AH16275

[3] Reichert A, Jacobs R. The impact of waiting time on patient outcomes: evidence from early intervention in psychosis services in England. Health Econ. 2018;27(11):1772–87. 30014544 10.1002/hec.3800 PMC6221005

[4] Stute M, et al. Process to establish 11 primary contact allied health pathways in a public health service. Aust Health Rev. 2018;42(3):258–65. 28483033 10.1071/AH16206

[5] Moretto N, et al. A uniform data set for determining outcomes in allied health primary contact services in Australia. Aust J Prim Health. 2020;26(1):58–69. 31954431 10.1071/PY18104

[6] Ashmore K, et al. Triage of knee pain by an Extended Scope Physiotherapist (ESP) in an orthopaedic clinic: a clinical audit. Physiother Pract Res. 2014;35(1):25–32.

[7] Stute M, et al. Allied health primary contact services: results of a 2-year follow-up study of clinical effectiveness, safety, wait times and impact on medical specialist out-patient waitlists. Aus Health Rev. 2021;45(3):344–52. 10.1071/AH19225 33271059

[8] Mutsekwa RN, et al. Dietitian first gastroenterology clinic: an initiative to reduce wait lists and wait times for gastroenterology outpatients in a tertiary hospital service. Frontline Gastroenterol. 2019;10(3):229–35. 31281623 10.1136/flgastro-2018-101063 PMC6583570

[9] Mutsekwa RN, et al. Patient preferences for attributes that characterise alternative models of care in gastroenterology: a discrete choice experiment. Patient. 2023;16(2):165–77. 36637751 10.1007/s40271-022-00609-4

[10] Mutsekwa RN, et al. Role substitution of specialist medical doctors with allied-health professionals: a qualitative exploration of patients’ experiences and perceptions of healthcare quality. J Eval Clin Pract. 2022;28(6):1096–105. 35470945 10.1111/jep.13691

[11] Kularatna S, et al. Cancer survivor preferences for models of breast cancer follow-up care: selecting attributes for inclusion in a discrete choice experiment. Patient. 2023;16(4):371–83. 37213062 10.1007/s40271-023-00631-0 PMC10201515

[12] Webb EJD, et al. Attribute selection for a discrete choice experiment incorporating a best-worst scaling survey. Val Health. 2021;24(4):575–84. 10.1016/j.jval.2020.10.025 33840436

[13] Hollin IL, et al. Best-worst scaling and the prioritization of objects in health: a systematic review. Pharmacoeconomics. 2022;40(9):883–99. 35838889 10.1007/s40273-022-01167-1 PMC9363399

[14] Sypek MP, et al. Healthcare professional and community preferences in deceased donor kidney allocation: a best-worst scaling survey. Am J Transplant. 2022;22(3):886–97. 34839582 10.1111/ajt.16898

[15] Arksey H, O’Malley L. Scoping studies: towards a methodological framework. Int J Social Res Methodol. 2005;8(1):19–32.

[16] Louviere J, et al. An introduction to the application of (case 1) best–worst scaling in marketing research. Int J Res Mark. 2013;30(3):292–303.

[17] Furlan R, Turner G. Maximum difference scaling: exploring the impact of design elements on results. Int J Mark Res. 2014;56(3):367–85.

[18] Cheung KL, et al. Comparison of statistical analysis methods for object case best-worst scaling. J Med Econ. 2019;22(6):509–15. 30482068 10.1080/13696998.2018.1553781

[19] Ozdemir S, et al. An overview of data collection in health preference research. Patient (2024). 10.1007/s40271-024-00695-6 38662323

[20] de Bekker-Grob EW, et al. Sample size requirements for discrete-choice experiments in healthcare: a practical guide. Patient. 2015;8(5):373–84. 25726010 10.1007/s40271-015-0118-z PMC4575371

[21] Watson V, Becker F, de Bekker-Grob E. Discrete choice experiment response rates: a meta-analysis. Health Econ. 2017;26(6):810–7. 27122445 10.1002/hec.3354

[22] R Core Team. R: A language and environment for statistical computing; 2024. https://www.R-project.org/.

[23] Sarrias M, Daziano R. Multinomial logit models with continuous and discrete individual heterogeneity in R: the gmnl package. J Stat Softw. 2017;79(2):1–46.30220889

[24] Flynn T, Marley A. Best-worst scaling: theory and methods. In: Hess S, Daly A, editors. Handbook of choice modelling. Cheltenham: Edwin Elgar; 2014. p. 178–201.

[25] Hart A, Dixon A. Sonographer role extension and career development: a review of the evidence. Ultrasound. 2008;16(1):31–5.

[26] Zeitoun H, et al. Assessment of a direct referral hearing aid clinic. Br J Audiol. 1995;29(1):13–21. 8580892 10.3109/03005369509086581

[27] Bath B. Biopsychosocial evaluation of a spinal triage service delivered by physiotherapists in collaboration with orthopaedic surgeons. Saskatchewan: University of Saskatchewan; 2012. 10.3138/ptc.2011-29 PMC3484906 23997390

[28] Napier C, et al. A physiotherapy triage service for orthopaedic surgery: an effective strategy for reducing wait times. Physiother Can. 2013;65(4):358–63. 24396164 10.3138/ptc.2012-53 PMC3817883

[29] Razmjou H, et al. Evaluation of an advanced-practice physical therapist in a specialty shoulder clinic: diagnostic agreement and effect on wait times. Physiother Can. 2013;65(1):46–55. 24381382 10.3138/ptc.2011-56 PMC3563377

[30] Burrows L, et al. Independent prescriber physiotherapist led balance clinic: the Southport and Ormskirk pathway. J Laryngol Otol. 2017;131(5):417–24. 28202097 10.1017/S0022215117000342

[31] Peck F, Kennedy S, McKirdy L. The introduction of practitioner-led hand clinics in South Manchester. Br J Hand Ther. 2001;6(2):41–4.

[32] Smyth C, et al. Physiotherapist-led triage of patients with thoracic spine pain in a musculoskeletal assessment clinic: a service evaluation of activity and outcomes. Physiother Pract Res. 2019;40(2):145–53.

[33] Hussenbux A, et al. Intermediate care pathways for musculoskeletal conditions–are they working? A systematic review. Physiotherapy. 2015;101(1):13–24. 25442485 10.1016/j.physio.2014.08.004

[34] Saxon RL, Gray MA, Oprescu FI. Reducing geriatric outpatient waiting times: Impact of an advanced health practitioner. Australas J Ageing. 2018;37(1):48–53. 29044886 10.1111/ajag.12459

[35] Fennelly O, et al. Advanced practice physiotherapy-led triage in Irish orthopaedic and rheumatology services: national data audit. BMC Musculoskelet Disord. 2018;19(1):181. 29859072 10.1186/s12891-018-2106-7 PMC5984783

[36] Daker-White G, et al. A randomised controlled trial. Shifting boundaries of doctors and physiotherapists in orthopaedic outpatient departments. J Epidemiol Community Health. 1999;53(10):643–50. 10616677 10.1136/jech.53.10.643 PMC1756791

[37] Samsson KS, et al. Effects on health and process outcomes of physiotherapist-led orthopaedic triage for patients with musculoskeletal disorders: a systematic review of comparative studies. BMC Musculoskelet Disord. 2020;21(1):1–20. 10.1186/s12891-020-03673-9 PMC7548042 33038935

[38] Burn D, Beeson E. Orthopaedic triage: cost effectiveness, diagnostic/surgical and management rates. Clin Gov Int J. 2014;19(2):126–36.

[39] Williams AH, et al. Impacts of advanced physiotherapy: a narrative literature review. N Z J Physiother. 2019;47(3):150–9.

[40] Brennen R, Sherburn M, Rosamilia A. Development, implementation and evaluation of an advanced practice in continence and women’s health physiotherapy model of care. Aus N Z J Obstet Gynaecol. 2019;59(3):450–6. 10.1111/ajo.12974 30957894

[41] Marks D, et al. Substitution of doctors with physiotherapists in the management of common musculoskeletal disorders: a systematic review. Physiotherapy. 2017;103(4):341–51. 28801031 10.1016/j.physio.2016.11.006

[42] McEvoy C, et al. Triage for patients with spinal complaints: a systematic review of the literature. Physiotherapy Res Int. 2017. 10.1002/pri.1639. 10.1002/pri.1639 26343816

[43] Belthur MV, Clegg J, Strange A. A physiotherapy specialist clinic in paediatric orthopaedics: is it effective? Postgrad Med J. 2003;79(938):699–702. 14707248 PMC1742895

[44] Weale AE, Bannister GC. Who should see orthopaedic outpatients—physiotherapists or surgeons? Ann R Coll Surg Engl. 1995;77(2 Suppl):71–3. 7574300

[45] Moloney A, et al. A 6-month evaluation of a clinical specialist physiotherapist’s role in a fracture clinic. Physiother Ireland. 2009;30(1):8–15.

[46] Robarts S, et al. A framework for the development and implementation of an advanced practice role for physiotherapists that improves access and quality of care for patients. Healthc Q. 2008;11(2):67–75. 18362523 10.12927/hcq.2008.19619

[47] Nucifora J, et al. Outcomes of a physiotherapy-led pelvic health clinic. Aus N Z Cont J. 2018;24(2):43–50.

[48] Ryan D, Pelly F, Purcell E. The activities of a dietitian-led gastroenterology clinic using extended scope of practice. BMC Health Serv Res. 2016;16(1):604. 27769223 10.1186/s12913-016-1845-0 PMC5073884

[49] Kasbekar AV, et al. Development of a physiotherapy-led balance clinic: the Aintree model. J Laryngol Otol. 2014;128(11):966–71. 25311108 10.1017/S0022215114002060

[50] Murphy MT, Radovanovic J. Patient satisfaction with physiotherapists is not inferior to surgeons in an arthroplasty review clinic: non-inferiority study of an expanded scope model of care. Aus Health Rev. 2021;45(1):104–9. 10.1071/AH19217 33342461

[51] Flynn TN, et al. Best–worst scaling: What it can do for health care research and how to do it. J Health Econ. 2007;26(1):171–89. 16707175 10.1016/j.jhealeco.2006.04.002

[52] Karthikeyan A, et al. Real-world outcomes of allied health professional-led clinic model for assessing and monitoring ocular melanocytic lesions. Eye. 2021;35(2):464–9. 32317788 10.1038/s41433-020-0873-5 PMC8026979

