# Supplementary figures and images for: Using Best–Worst Scaling Survey to Investigate the Relative Importance of Attributes Associated with Public Hospital Outpatient Appointments
Source: Patient. 2025 Feb 26;18(3):237–47. doi: 10.1007/s40271-025-00732-y (PMC11985688; doi:10.1007/s40271-025-00732-y)

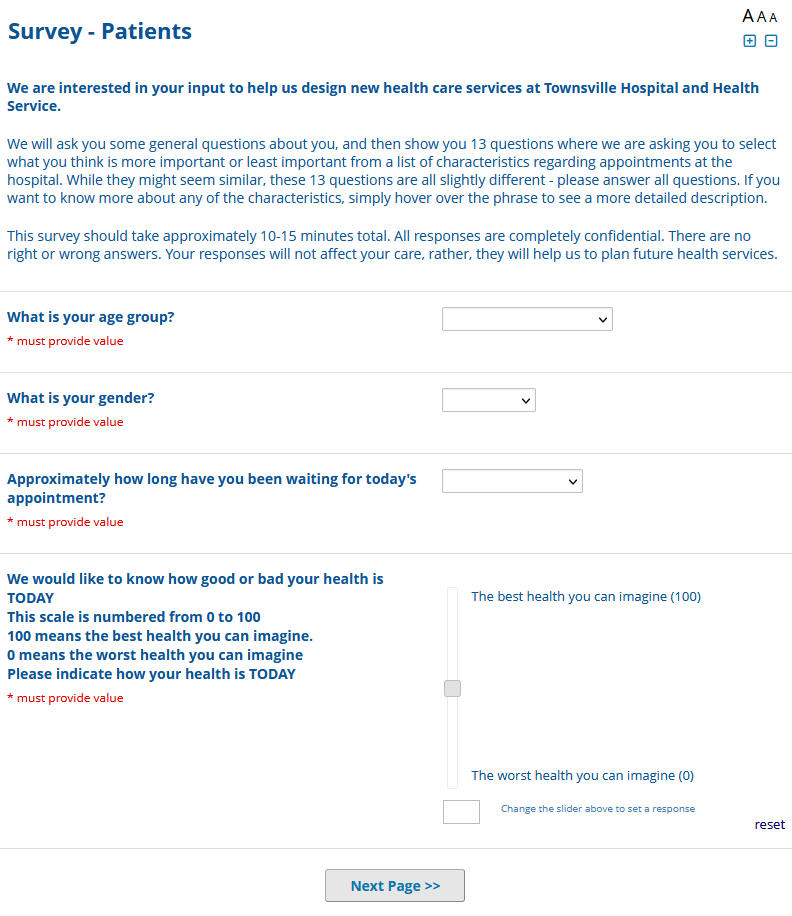

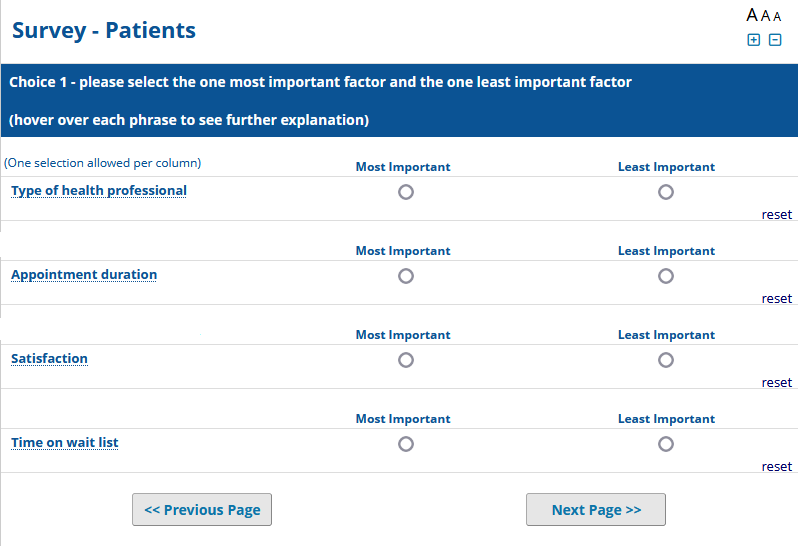

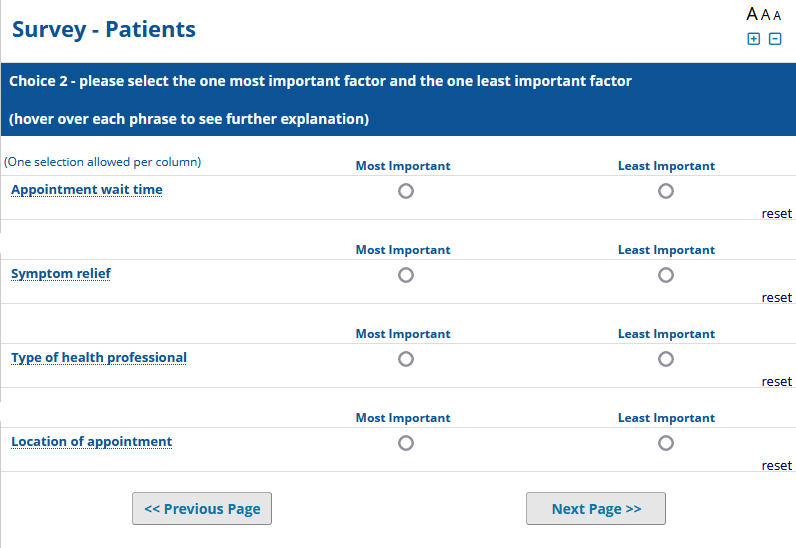

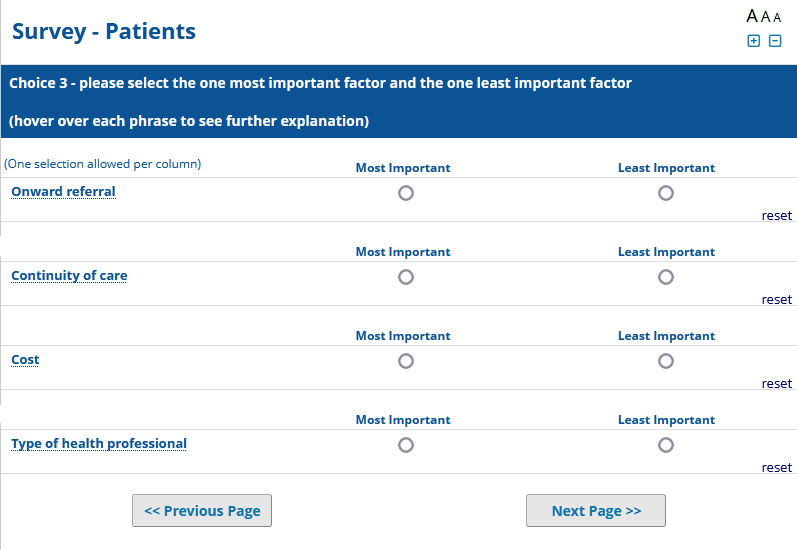

Supplement: Supplementary file 2 — Supplementary file2 (DOCX 261 kb) [file 40271_2025_732_MOESM2_ESM.docx]
