# Supplementary material for: Using Best–Worst Scaling Survey to Investigate the Relative Importance of Attributes Associated with Public Hospital Outpatient Appointments
Source: Patient. 2025 Feb 26;18(3):237–47. doi: 10.1007/s40271-025-00732-y (PMC11985688; doi:10.1007/s40271-025-00732-y)
Supplement: Supplementary file 3 — Supplementary file3 (DOCX 24 kb) [file 40271_2025_732_MOESM3_ESM.docx]

**Legend for Supplement 1.**

| **Column** | **Abbreviations** |
| --- | --- |
| NHMRC Levels of Evidence | I: A systematic review of Level II studies, II: A randomised controlled trial, III-1: A pseudo-randomised controlled trial, III-2: A comparative study with concurrent controls, III-3: A comparative study without concurrent controls, IV: Case studies with either post-test or pre-test/post-test outcomes. |
| Allied Health Discipline (AH) Discipline | PT – physiotherapist, OT – occupational therapist, Diet – dietitian, Pod – podiatrist, Aud – Audiologist, SP – speech pathologist, Opt – optometrist, Radiog – radiographer |
| AH Experience (Exp) | Ext – extended, Exp – expanded, Adv practice – advanced practice |
| Medical discipline | Ortho – orthopaedic surgeon, Neuro – Neurologist, Neuro surg – neuro surgeon, Urogyn – urogynacologist, Rheum – rheumatologist, ENT – ear nose throat specialist, Opthal – ophthalmologist, Radiol – radiologist, Geri – geriatrician, Gastro – gastroenterology, Otol – Otolaryngology |
| Substitution model | Pt – patient, Pts – patients, AH – allied health, CP cerebral palsy, Ax – assessment, Mgt – management, Ix – investigations, Appt – appointment, Cat – category, HP – health practitioner, GP – general practitioner, |
| Patient preference | NS – not specified, |
| Impact on wait time | Av – average, imple – implementation, |
| Outcome | N – number, med itn – medical intervention, DNA – did not attend, WOMAC – West Ontario and McMaster Universities’ Osteoarthritis Index, KOOS – Knee Injury and Osteoarthritis Outcome Score, HOOS – Hip Dysfunction and Osteoarthritis Outcome Score, HRQoL – Health related quality of life, PREM – patient reported outcome measure, CSQ - Client Satisfaction Questionnaire, VSQ – Visit Specific Satisfaction Questionnaire, MOPC – Measure of Processes of Care, APFQ – Australian Pelvic Floor Questionnaire, AQol – Assessment of Quality of Life, GROC – global rating of change, VAS – visual analogue scale, EQ5D – EurolQuol 5 dimension, QOL – quality of life, |
